# Immunohistochemical Evaluation of Integrin αvβ6 Expression in Gastric and Gastroesophageal Junction Adenocarcinoma as a Histopathology-Driven Biomarker for Integrin αvβ6-Targeted Radiotheranostics

**DOI:** 10.3390/cancers18142231

**Published:** 2026-07-11

**Authors:** Muin Tuffaha, Wael Hananeh, Michael Starke

**Affiliations:** 1 Institute of Pathology, Medical University Lausitz-Carl-Thiem, 03048 Cottbus, Germany; m.tuffaha@mul-ct.de; 2 Department of Veterinary Pathology and Public Health, Faculty of Veterinary Medicine, Jordan University of Science and Technology, Irbid 22110, Jordan; 3 Nuclear Medicine, Medical University Lausitz-Carl-Thiem, 03048 Cottbus, Germany; m.starke@mul-ct.de

**Keywords:** gastric adenocarcinoma, gastroesophageal junction adenocarcinoma, integrin αvβ6, immunohistochemistry, radiotheranostics, 68Ga-Trivehexin, 177Lu, 161Tb, 225Ac

## Abstract

Background: Gastric and gastroesophageal junction adenocarcinomas remain major causes of cancer- related mortality worldwide. Although perioperative chemotherapy, HER2-directed therapy, Claudin-18.2 (CLDN18.2)-targeted treatment, immune checkpoint inhibition, and anti-angiogenic therapy have improved outcomes in selected patients, clinically actionable targets are absent, heterogeneous, or lost in many high-grade, diffuse, poorly cohesive, signet-ring-cell micropapillary, hepatoid, and sarcomatoid carcinomas. Additional histopathology-assessable biomarkers that can support both molecular imaging and targeted therapy are of considerable clinical interest. Methods: Integrin αvβ6 is an epithelial-specific adhesion receptor that is minimally expressed in most normal adult tissues but becomes markedly upregulated during epithelial carcinogenesis, tissue remodeling, invasion, and activation of transforming growth factor-β signaling. This retrospective histopathological and immunohistochemical study evaluated integrin αvβ6 expression by immunohistochemistry in 53 formalin-fixed paraffin-embedded diagnostic specimens of untreated primary gastric or gastroesophageal junction adenocarcinoma. Tumors were classified according to the WHO Classification of Tumors of the Digestive System, 5th edition. Membranous integrin αvβ6 staining intensity and the percentage of positive tumor cells were recorded, and an H-score was calculated. Expression patterns were compared descriptively with histological subtype, differentiation grade, HER2 status, and CLDN18.2 expression. Results: Integrin αvβ6 immunoreactivity was detected in 49 of 53 tumors (92.5%), the clinically more relevant moderate to strong expression (H-score > 100) was present in 27 of 53 cases (50.9%), and strong expression was present in 13 of 53 cases (24.5%). Expression was not confined to conventional intestinal-type tumors; among carcinomas containing a signet-ring-cell component, 14 of 16 assessable cases (87.5%) showed integrin αvβ6 positivity, with 8 of 16 (50.0%) showing moderate to strong staining. By comparison, HER2 positivity was identified in 4 of 53 cases (7.5%; 4 of 52 assessable cases, 7.7%), and clinically relevant CLDN18.2 positivity, defined as moderate-to-strong membranous staining in at least 75% of tumor cells, was present in 7 of 38 assessable cases (18.4%). Conclusions: Integrin αvβ6 is frequently expressed in gastric and gastroesophageal junction adenocarcinoma, with moderate to strong expression in approximately half of all cases and in half of signet ring cell-containing tumors, including aggressive histological subtypes that often lack established therapeutic targets. The precise H-Score threshold for theranostic eligibility remains to be determined. The findings support integrin αvβ6 as a promising biomarker for histopathology-based screening, PET-based whole-body target assessment, and future targeted radionuclide therapy. Integration of immunohistochemistry and integrin αvβ6- targeted PET imaging will be essential to validate integrin αvβ6 as a candidate biomarker and to optimize radionuclide selection in gastric cancer, particularly with moderate to strong expression.

## Simple Summary

Many patients with stomach or gastroesophageal junction cancer lack effective targeted treatments, as current biomarker tests for HER2 and claudin-18.2 are often negative, particularly in the more aggressive tumor types. The objective of this study was to examine whether the cell-surface protein integrin αvβ6 could serve as a new biomarker candidate for diagnosis and/or targeted therapy. We examined tumor samples from 53 patients and found that moderate-to-strong integrin αvβ6 expression—the level most likely to be therapeutically relevant—was present in 50.9% of cases, with strong expression in 24.5%. While any degree of detectable expression was present in 92.5% of tumors, the lower—level staining (H-score ≤ 100) observed in a subset of these cases may not be sufficient for effective radioligand binding. Importantly, integrin αvβ6 expression was retained in aggressive histological subtypes, including 87.5% (14 of 16 assessable cases) of signet-ring cell carcinomas; of these, moderate-to-strong expression was seen in 56.3% (9 of 16) at the whole- tumor level and 50.0% (8 of 16) specifically within the signet-ring-cell component. Since integrin αvβ6 is located on the surface of the tumor cell, it can be visualized via specialized imaging and targeted with radioactive drugs. Together, these findings suggest that integrin αvβ6 may serve as a candidate biomarker for identifying patients who could benefit from a novel class of personalized targeted radionuclide therapies in modern nuclear medicine, thereby expanding treatment options for patients with limited or no effective therapeutic alternatives. However, the precise H-score threshold that ensures adequate therapeutic efficacy has yet to be prospectively validated.

## 1. Introduction

Gastric cancer remains one of the most common malignancies and one of the leading causes of cancer-related death worldwide. The most recent GLOBOCAN-based global estimates reported approximately 968,000 new stomach cancer cases and 660,000 deaths in 2022, ranking stomach cancer fifth for incidence and fifth for mortality among all cancers [1]. Despite improvements in multimodal therapy, advanced gastric and gastroesophageal junction adenocarcinomas remain clinically challenging because of pronounced histological, molecular, and spatial heterogeneity.

Conventional chemotherapy regimens show only modest efficacy in many patients, particularly in diffuse-type gastric adenocarcinomas, including poorly cohesive carcinoma and signet-ring cell carcinoma. Biomarker-directed therapy in gastric and gastroesophageal junction adenocarcinoma currently relies mainly on HER2, CLDN18.2, PD-L1, microsatellite instability/mismatch repair deficiency, and selected rare molecular alterations. An HER2-directed therapy is established in HER2-positive advanced disease; however, HER2 positivity is enriched in intestinal-type, cohesive, and gastroesophageal junction tumors and is less frequent in diffuse, poorly cohesive, and signet-ring-cell carcinomas [2,3]. In the ToGA screening cohort, HER2 positivity was reported in 22.1% of analyzed tumors, with substantially higher frequency in intestinal-type than diffuse-type disease [3].

CLDN18.2 has recently emerged as a clinically relevant therapeutic target in HER2-negative advanced gastric and gastroesophageal junction adenocarcinoma. The phase III SPOTLIGHT and GLOW trials demonstrated improved progression-free and overall survival when zolbetuximab was combined with chemotherapy in patients whose tumors showed moderate-to-strong CLDN18.2 membranous staining in at least 75% of tumor cells [4,5]. Nevertheless, CLDN18.2 expression may be heterogeneous, may decline in poorly differentiated or poorly cohesive components, and is not present at clinically actionable levels in all patients [6,7,8,9]. Therefore, additional biomarkers are required, particularly for HER2-negative and CLDN18.2-low tumors and for histological subtypes with aggressive infiltrative growth.

Integrins are heterodimeric transmembrane receptors composed of α and β subunits that mediate cell–cell and cell–extracellular matrix interactions. Through bidirectional signaling, they regulate adhesion, migration, proliferation, differentiation, survival, apoptosis, extracellular matrix remodeling, and immune interactions [9,10]. In cancer, dysregulated integrin signaling contributes to invasion, epithelial- mesenchymal plasticity, metastasis, angiogenesis, and resistance to therapy [9,10]. Within the integrin family, integrin αvβ6 has attracted considerable attention due to its highly restricted expression pattern. The β6 subunit, encoded by ITGB6, pairs with the αv subunit to form αvβ6. This integrin binds latency-associated peptide and activates latent transforming growth factor-β, linking epithelial remodeling to fibrosis, immune suppression, invasion, and tumor progression [11,12]. Unlike many other integrins, integrin αvβ6 is predominantly expressed on epithelial cells and is largely absent in most normal adult tissues under physiological conditions. However, its expression is markedly upregulated during epithelial remodeling processes such as wound healing, fibrosis, inflammation, and carcinogenesis [13]. This selective expression profile has made αvβ6 a molecule of increasing interest in cancer biology and targeted therapy research [14].

Recent gastric cancer data further support the relevance of integrin αvβ6 [15]. Yu et al. [16] reported that integrin αvβ6 expression was associated with tumor size, pathological grade, nodal stage, clinical stage, and survival, and experimental data suggested that ITGB6 may promote proliferation, migration, and invasion partly through Rac1-related mechanisms. Hou et al. [17] provided a comprehensive review characterizeing integrins, particularly integrin αvβ6, in gastrointestinal cancer, as potentially useful biomarkers and therapeutic targets because they exhibit altered levels of expression that are regulated at transcriptional, post-transcriptional, and post-translational levels. These findings strengthen the rationale for evaluating integrin αvβ6 as a histopathology-accessible biomarker and potential therapeutic target in gastric adenocarcinoma. Integrin αvβ6 is an epithelial-specific receptor but has low expression levels in normally developed adult tissue [18]. However, this receptor is highly expressed in epithelial cancers and is involved in invasion, activation of TGF-β, and the progression of tumors. Therefore, this receptor differs from current biomarkers, including HER2 and CLDN18.2, as this receptor may have greater expression patterns across many subtypes of aggressive cancers, such as signet-ring cell carcinomas, in which HER2 and CLDN18.2 are frequently either negative or heterogeneous, making integrin αvβ6 an attractive, more likely broadly expressed target for radiotheranostics in an area of significant unmet medical need.

The restricted expression profile and tumor-associated upregulation of αvβ6 have led to the development of radiolabeled ligands for molecular imaging and therapy. αvβ6-directed PET agents, including 68Ga-Trivehexin, have demonstrated favorable tumor visualization in αvβ6-expressing malignancies, particularly pancreatic, head-and-neck and breast cancers [14,19,20]. Preclinical αvβ6- targeted radiotheranostic platforms using gallium-68 (^68^Ga) for imaging and lutetium-177 (^177^Lu) for therapy have also been reported [21,22]. More recently, the first clinical αvβ6-targeted radioligand therapy using ^177^Lu-Therahexin has been described, supporting early clinical feasibility of this theranostic concept [23].

Therapeutic radionuclide selection is central to treatment design. β-emitting radionuclides such as ^177^Lu and terbium-161 (^161^Tb) generate a crossfire effect that can irradiate neighboring tumor cells with low or absent target expression. In contrast, α-emitting radionuclides such as ^225^Ac deliver high-linear- energy-transfer radiation over a shorter range and therefore depend more strongly on direct targeting of individual tumor cells. ^161^Tb is of particular interest because it emits β-particles together with conversion and Auger electrons, potentially increasing local absorbed dose around directly targeted cells and microscopic tumor deposits [24,25]. These early clinical findings highlight the emerging potential of integrin αvβ6 as a novel theranostic target in oncology.

This study aimed to evaluate immunohistochemical integrin αvβ6 expression in untreated primary gastric and gastroesophageal junction adenocarcinoma and to assess its potential role as a histopathology-driven candidate biomarker for αvβ6-targeted PET imaging and radionuclide therapy. We further compared integrin αvβ6 expression with HER2 and CLDN18.2 expression and considered how tumor architecture may influence radionuclide selection.

Although integrin αvβ6 is generally considered minimally expressed or absent in most adult quiescent tissues, physiological expression can occur in certain tissues, including the gastrointestinal mucosa. The gastrointestinal epithelium exists in a state of persistent physiological activity characterized by rapid epithelial turnover, continuous regeneration, and tightly regulated immune surveillance rather than true cellular quiescence [26]. This baseline expression is relevant when evaluating integrin αvβ6-targeted imaging or therapeutic approaches in gastric cancer and warrants careful assessment in forthcoming biodistribution and dosimetry investigations.

## 2. Materials and Methods

### 2.1. Study Design, Case Selection, and Tissue Samples

This retrospective observational study used archived formalin-fixed paraffin-embedded tissue specimens from patients with histologically confirmed untreated primary gastric or gastroesophageal junction adenocarcinoma. All cases were retrieved from the Institute of Pathology’s archive at the Medical University Lausitz-Carl-Thiem, Germany, covering the period from January 2024 to March 2026. A total of 53 cases were included. All tissue samples consisted of all available diagnostic gastric biopsy specimens obtained during gastroscopy. Cases with poor tissue preservation, insufficient tumor tissue, recurrent or metastatic tumors without available primary tumor tissue, or incomplete clinicopathological information were excluded.

### 2.2. Histopathological Evaluation

Hematoxylin and eosin-stained slides were reviewed by experienced pathologists to confirm the diagnosis and evaluate histological subtype, differentiation grade, signet-ring-cell component, and available clinicopathological parameters. Tumors were classified according to the WHO Classification of Tumours of the Digestive System, 5th edition [27,28]. Pathology terminology was harmonized as gastric adenocarcinoma, gastroesophageal junction adenocarcinoma, intestinal-type adenocarcinoma, poorly cohesive carcinoma, signet-ring-cell carcinoma, and mixed adenocarcinoma as appropriate. Discrepant diagnoses were resolved by consensus review.

### 2.3. Immunohistochemistry

Serial 1-µm sections were cut from formalin-fixed paraffin-embedded tissue blocks and mounted on positively charged glass slides. After deparaffinization and rehydration, heat-induced antigen retrieval was performed using Tris-EDTA buffer, pH 9.0. Endogenous peroxidase activity was blocked before incubation with the primary antibody.

Integrin αvβ6 immunohistochemistry was performed using a rabbit monoclonal antibody against integrin β6/αvβ6 (clone E4M9P; Cell Signaling Technology, Danvers, MA, USA) at a dilution of 1:200. Immunoreactivity was visualized using a two-step horseradish peroxidase/diaminobenzidine detection system, followed by hematoxylin counterstaining. To reduce inter-assay variability, staining was performed under standardized conditions using identical reagents and an automated staining platform within a single experimental run. Photomicrographs were captured, and the total magnifications are stated in the corresponding figure legends.

**Figure 1:**
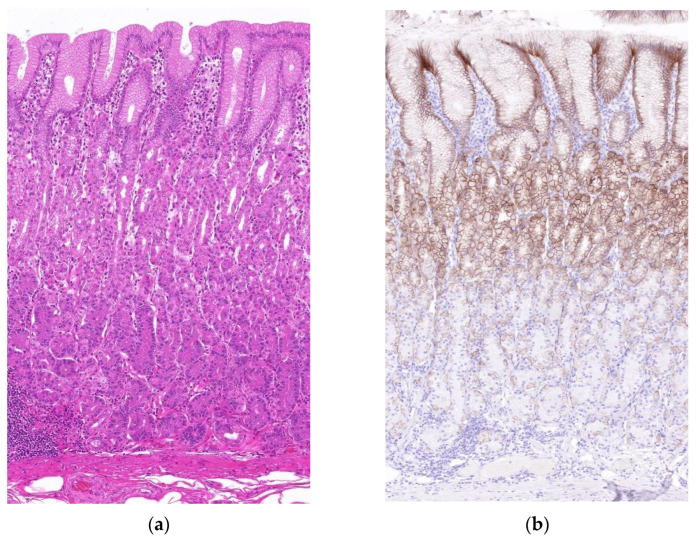
Histologic section of normal stomach. (**a**) Full thickness of normal human gastric mucosa, including the muscularis mucosae. H&E 40×. (**b**) Immunohistochemical staining for integrin αvβ6 demonstrates strong expression in the middle portion of the gastric glands, particularly within the neck and isthmus regions. Expression decreases toward the glandular base and, to a lesser extent, toward the foveolar epithelium. IHC 40×.

**Figure 2:**
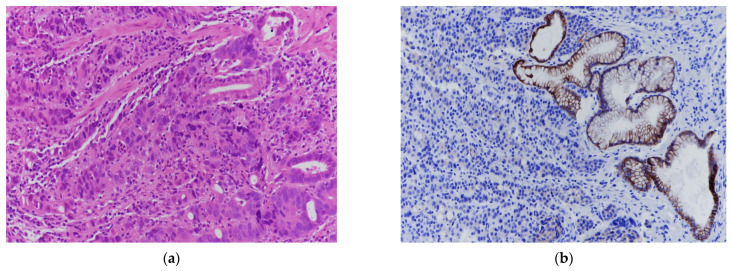
(**a**) Intestinal-type gastric adenocarcinoma. H&E. 200×. (**b**) Only a few tumor cells (<5%) show very weak integrin αvβ6 expression, whereas the adjacent gastric mucosa exhibited strong membranous expression. IHC. 200×.

Adjacent non-neoplastic gastric mucosa, when present, served as an internal positive control and demonstrated the expected compartmentalized epithelial staining pattern (Figure 1 and Figure 2). Negative controls were included in each staining run. After IHC slides were prepared, a pathologist performed a single-blind evaluation of the slides and the scores were recorded. The scoring pathologist had no access to any clinicopathological data during slide assessment.

### 2.4. Evaluation of Immunohistochemical Staining

Integrin αvβ6 expression was evaluated semi-quantitatively by assessing membranous staining intensity and the estimated percentage of positive tumor cells. Staining intensity was scored as 0, negative; 1, weak; 2, moderate; and 3, strong. The percentage of positive tumor cells was recorded in the evaluated tumor compartment. An H-score was calculated by multiplying intensity by the percentage of positive tumor cells, resulting in a final score from 0 to 300. Mixed intensities were scored using the mean intensity value; for example, weak-to-moderate staining was scored as 1.5 and moderate-to-strong staining as 2.5. This convention was utilized solely for tumors in which two neighboring intensity grades were intermixed within the same membranous staining pattern and could not be allocated to a single discrete category; consequently, the resulting non-integer H-scores were preserved without rounding to maintain scoring resolution.

Cases were categorized as negative (H-score 0), weak/low expression (H-score 1–100), moderate expression (H-score 101–200), or strong/high expression (H-score 201–300) as previously described [29]. These categories are employed descriptively as a practical four-tier subdivision of the 0–300 H-score range; they do not denote a validated clinical threshold or a therapeutic-relevance benchmark. Furthermore, the H-score corresponding to a tumor that would be amenable to αvβ6-directed therapy remains to be determined. All HER2 and CLDN18.2 results were recorded from prior available diagnostic immunohistochemistry reports. CLDN18.2 clinically relevant positivity was defined as moderate-to-strong membranous staining in at least 75% of tumor cells, corresponding to the criterion used in the pivotal zolbetuximab trials [4,5]. The intensity symbols (+/++/+++) and the H-score categories are different scoring systems and should not be read as equivalent. The symbols record raw staining intensity, whereas the H-score category is derived from the calculated intensity × percentage product. For the CLDN18.2 column, entries given as a percentage alone (without an accompanying intensity symbol) correspond to cases for which staining intensity was not specified in the original diagnostic report; intensity is shown where it was recorded as part of the present study’s evaluation.

### 2.5. Statistical Analysis

Categorical variables are presented as numbers and percentages. Wilson score method was used to calculate the 95% confidence intervals (CIs) for proportions. Associations between integrin αvβ6 H-score category (or αvβ6 status: positive/negative) and histological subtype (presence versus absence of signet-ring-cell component), HER2 status, and clinically relevant CLDN18.2 status were evaluated using Fisher’s exact test on the corresponding case-level contingency tables. Because HER2, CLDN18.2, and integrin αvβ6 were each evaluated within the same tumors, the relative frequency of positivity between markers in the cohort was additionally compared using the exact (binomial) McNemar test for paired proportions. All tests were two-sided, with *p* < 0.05 denoting statistical significance. Statistical analyses were performed using GraphPad Prism version 11 for Windows, GraphPad Software, Boston, MA, USA.

## 3. Results

A total of fifty-three diagnostic gastric or gastroesophageal junction biopsy specimens from patients with histologically confirmed primary adenocarcinoma were evaluated for integrin αvβ6 expression by immunohistochemistry. Adequate tumor tissue was available in all cases. Normal gastric mucosa, serving as an internal positive control, was present in all biopsies (Figure 1).

Integrin αvβ6 is predominantly expressed on malignant epithelial plasma membranes, with only minimal or rare staining of cytoplasmic compartments and very little or no staining in the stromal compartments. Staining intensities for integrin αvβ6 varied considerably among tumor types and regions. This finding indicates intratumoral heterogeneity of integrin αvβ6 expression. Such heterogeneity may be relevant in a diagnostic context. It may also influence future radiotheranostic use, because spatial variation in target expression could influence both the amount of tracer accumulation in tissue and the distribution of therapeutic doses administered. Table 1 summarizes the clinicopathological features and biomarker expression profiles (integrin αvβ6, HER2 and CLDN18.2) for all 53 cases of gastric and gastroesophageal junction adenocarcinoma. AEG cases were classified by Siewert type, where documented.

### 3.1. Integrin αvβ6 Expression in the Overall Cohort

**Figure 3:**
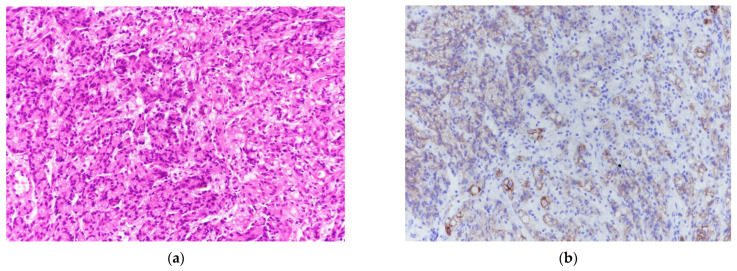
(**a**) Intestinal-type gastric adenocarcinoma. H&E. 200×; (**b**) Approximately 75% of tumor cells show moderate to strong membranous integrin αvβ6 expression. IHC. 200×.

**Figure 4:**
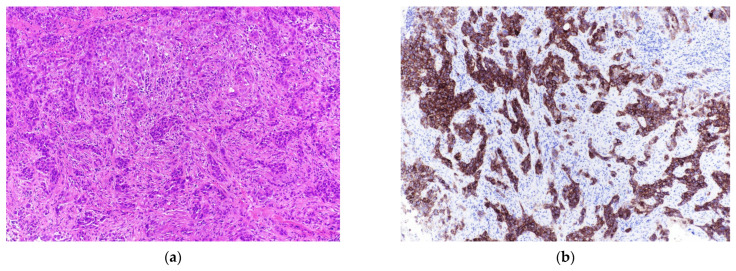
(**a**) Intestinal-type gastric adenocarcinoma. H&E. 40×; (**b**) Approximately 90% of tumor cells show moderate to strong membranous integrin αvβ6 expression. IHC. 40×.

Integrin αvβ6 expression was detected in 49 of 53 tumors (92.5%, 95% CI: 82.1–97.0%). Four cases were negative. Moderate or strong expression was present in 27 of 53 cases (50.9%, 95% CI: 37.9–63.9%), and strong expression was observed in 13 of 53 cases (24.5%, 95% CI: 14.9–37.6%) (Figure 2, Figure 3 and Figure 4). These results indicate that integrin αvβ6 is broadly expressed across the cohort and is present at potentially relevant levels in a substantial subset of tumors.

### 3.2. Expression According to Histological Subtype

**Figure 5:**
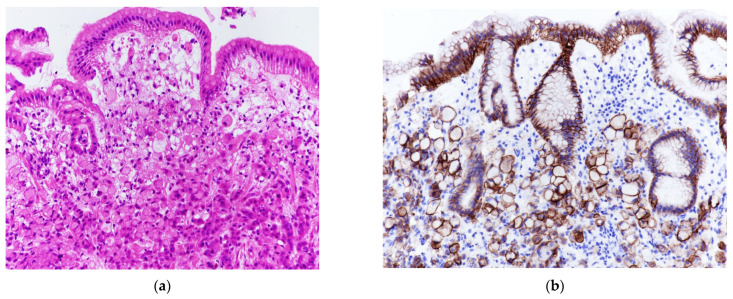
(**a**) Poorly cohesive signet-ring-cell gastric adenocarcinoma. H&E. 400×; (**b**) Approximately 90% of tumor of signet-ring- cells show strong membranous integrin αvβ6 expression. IHC. 400×.

Integrin αvβ6 expression was observed not only in intestinal-type adenocarcinoma but also in poorly cohesive and signet-ring-cell-containing tumors. Among tumors containing a signet-ring-cell component, 14 of 16 assessable cases (87.5%, 95% CI: 64.0–96.5%) were positive (Figure 5). This finding is particularly relevant because poorly cohesive carcinoma and signet-ring-cell carcinoma are frequently associated with infiltrative growth, peritoneal dissemination, reduced HER2 positivity, and heterogeneous expression of other actionable targets.

### 3.3. Comparison with HER2 and CLDN18.2

HER2 positivity was identified in 4 of 53 cases (7.5%, 95% CI: 3.0–17.9%). This proportion is lower than the approximately 15–25% reported in many gastric and gastroesophageal junction adenocarcinoma series and lower than the 22.1% positivity rate reported in the ToGA screening dataset [3]. The difference is likely related to the composition of the present cohort, which was enriched for high-grade and signet-ring-cell-containing tumors. In keeping with published data showing that HER2 positivity is more frequent in intestinal-type tumors and less frequent in diffuse-type tumors, none of the signet-ring-cell-containing tumors in this cohort was HER2-positive [2,3].

CLDN18.2 expression was assessable in 38 of 53 cases. Any CLDN18.2 staining was observed in 17 of 38 assessable cases (44.7%). Clinically relevant CLDN18.2 positivity, defined as moderate-to-strong membranous staining in at least 75% of tumor cells, was present in 7 of 38 assessable cases (18.4%, 95% CI: 9.2–33.4%). This is lower than the 38.4% prevalence reported in the global SPOTLIGHT/GLOW screening population using the same expression cutoff [5]. Among tumors with a signet-ring-cell component, CLDN18.2 was assessable in 12 cases; 5 of 12 (41.7%) showed any staining, and 2 of 12 (16.7%) fulfilled the 75% moderate-to-strong criterion. These findings are compatible with recent pathology studies showing that CLDN18.2 expression in poorly cohesive carcinoma can be present but heterogeneous and diagnostically challenging [7,8].

In contrast, integrin αvβ6 expression was considerably more frequent than either HER2 or clinically relevant CLDN18.2 expression. Integrin αvβ6 was positive in 49/53 cases (92.5%), with moderate or strong H-score categories in 27/53 cases (50.9%) and strong expression in 13/53 cases (24.5%). Integrin αvβ6 expression was also retained in signet-ring cells in 14/16 tumors with a signet-ring-cell component (87.5%). Therefore, in this cohort, integrin αvβ6 showed broader expression across conventional and signet-ring-cell-containing gastric/gastroesophageal adenocarcinomas than HER2 or CLDN18.2, suggesting that integrin αvβ6 may represent a more consistently expressed biomarker in this histologically high-grade cohort. Table 2 summarizes the comparative expression profiles of HER2, CLDN18.2 and integrin αvβ6, highlighting the markedly broader expression of integrin αvβ6 compared with HER2 and clinically relevant CLDN18.2 expression. Paired comparison within the same cohort demonstrated that the statistically significant difference between the prevalence rates was supported by the results of an exact McNemar test considering integrin αvβ6 and HER2 (*n* = 52 assessable; *p* < 0.0001), and integrin αvβ6 and clinically relevant CLDN18.2 (*n* = 38 assessable; *p* < 0.0001). Cross-tabulations indicate that moderate or strong (H-score > 100) levels of integrin αvβ6 expression did not differ between tumors with and without a signet-ring-cell component (9/16 [56.3%] vs. 18/37 [48.6%]; Fisher’s exact test, *p* = 0.77) or by HER2 status (4/4 [100%] HER2-positive vs. 45/48 [93.7%] HER2-negative cases were integrin αvβ6-positive; *p* = 1.00) or by CLDN18.2 status (6/7 [85.7%] CLDN18.2-positive vs. 29/31 [93.5%] CLDN18.2-negative cases were integrin αvβ6-positive; *p* = 0.47). Accordingly, the lack of significant subgroup differences indicates that integrin αvβ6 positivity was consistently high across all histological subtypes and HER2/CLDN18.2 statuses and would suggest a broader and less subtype-restricted pattern of expression rather than a difference limited to a specific subgroup; however, these comparisons should be considered as hypothesis-generating due to the limited sizes of the subgroups evaluated.

## 4. Discussion

This histopathology-led study demonstrates frequent integrin αvβ6 expression in untreated gastric and gastroesophageal junction adenocarcinoma. The primary finding of this study is not only the high frequency of integrin αvβ6 expression, but also its widespread distribution across different histological patterns. This is clinically significant because current biomarker- directed therapies in gastric and gastroesophageal junction adenocarcinoma remain limited by subtype restriction, heterogenous expression, and incomplete patient eligibility, particularly for HER2 and CLDN18.2-directed therapies [30,31]. Although integrin αvβ6 is expressed in healthy gastric mucosa and some other tissues involved in remodeling, there is much greater evidence that integrin αvβ6 is more specific than other cancer biomarkers because it is significantly upregulated in tumors in comparison to the low or non-existent expression seen in healthy tissue(s) (92.5% tumor positivity). Additionally, integrin αvβ6 is known to exhibit high sensitivity in more aggressive forms of cancer. Thus, integrin αvβ6 shows superior expression levels when compared with HER2 (7.5% tumor positivity), and CLDN18.2 (18.4% high expression). While this cohort demonstrates the prevalence and distribution of integrin αvβ6 expression in tumor tissue, the presence of IHC data alone does not yield formal sensitivity or specificity estimates for diagnostic classification, as a quantified non-tumor comparator cohort was not included. Additional validation using integrin αvβ6- targeted PET imaging and dosimetry is required.

Integrin αvβ6 positivity was identified in 92.5% of tumors, with moderate or strong expression in approximately half of the cohort. The expression was assessed in signet-ring cell positive tumors and was found to be present in 87.5% of tumors that contained signet-ring cells; moderate-to-strong staining was present in 56.3% (9 of 16) at the whole-tumor level (tumor-compartment H-score > 100) and in 50.0% (8 of 16) specifically within the signet-ring-cell component. The presence of moderate to strong staining in a majority of these tumors indicates that they are a biologically significant subgroup of gastric tumors related to clinical behavior such as aggressiveness, infiltrative growth, and resistance to standard therapies. This finding is aligned with prior evidence that integrin αvβ6 is involved in epithelial remodeling, TGF-β activation, invasion, and tumor progression, and with reports linking integrin αvβ6 expression in gastric carcinoma to adverse clinicopathological parameters such as pathological grading and staging, and overall patient survival [32].

In addition, limited evidence of expression of established therapeutic targets such as HER2 in this subgroup supports the hypothesis that integrin αvβ6 is a tumor-related marker that has biological significance and therapeutic potential throughout the heterogeneous histomorphological spectrum of gastric adenocarcinoma [32]. From a biological perspective, the predominant membranous pattern of staining for integrin αvβ6 supports its use as a targeted ligand for targeted therapies, as integrin αvβ6 is located on the cell membrane and is available for binding of targeted therapeutics [11,33]. Furthermore, it is clear from the results of the study that routine diagnostic immunohistochemistry will allow for the identification of integrin αvβ6 as a potential biomarker for integrin αvβ6-directed radioimmunotherapeutics in gastric cancers. The membranous localization is particularly important for radiotheranostic development because integrin αvβ6-targeted PET tracers and radioligands require accessible cell-surface binding. Previous studies of αvβ6-directed PET imaging, including 68Ga-Trivehexin, and preclinical or early clinical αvβ6-targeted radioligand therapy platforms support the feasibility of targeting this receptor in epithelial malignancies [20,34,35].

Collectively, the cohort of patients studied here demonstrates a significant gap in currently available biomarkers for gastric cancer. For instance, HER2 positivity was infrequent among the studied cohort, reflecting the majority of patients in the cohort with signet-ring or poorly differentiated gastric cancer. Although CLDN18.2 staining was more prevalent than HER2 staining in this patient population, the majority of assessable tumors were found to be below the cutoff for clinically relevant trial eligibility for zolbetuximab therapy [36,37]. Conversely, integrin αvβ6 expression occurred widely across all assessed histological patterns, raising the hypothesis that integrin αvβ6-directed approaches could complement, rather than compete with, CLDN18.2- and HER2-directed therapy in tumors negative or low for those targets. This complementary potential has not been evaluated within this study and would necessitate prospective assessment. Therefore, these data do not indicate that integrin αvβ6 is a clinically validated treatment-selection biomarker. Rather, integrin αvβ6 exhibits a broad expression profile and is emerging as a strong candidate for additional clinical validations within HER2-negative, CLDN18.2-low, or histologically aggressive tumors.

These findings have important therapeutic implications. Therapies that have targets of both HER2 and CLDN18.2 have limited use because of the biology of their antigens and the biologic nature of the two antibody mechanisms used in the therapies. Integrin αvβ6-directed therapy (theranostics) utilizes the biologic properties of the radio-ligand itself and relies on various factors, such as the binding of the radioligand, retention of the radioligand by the tumor, absorbed dose, heterogeneity of the target tumor, radiosensitivity of the tumor, and the physics associated with the radionuclide itself. Detection of integrin αvβ6 may represent yet another opportunity for targeted therapeutics, particularly in HER2-negative, CLDN18.2- low, and histologically aggressive tumors.

Integrin αvβ6 is specifically expressed in normal epithelial cells with very low levels in most normal adult tissues, which has led to the hypothesis of a potential therapeutic window for integrin αvβ6-targeting radiotheranostics. However, the current study’s own internal positive control showed strong membranous staining, and this mucosal expression was not systemically scored. While physiological expressions exist in places such as the gastric mucosa and other selected organs (pituitary gland, choroid plexus) [11,33], integrin αvβ6 is frequently expressed in different tumors. The upregulation of integrin αvβ6 in adjacent non-neoplastic gastric mucosa indicates that a favorable therapeutic window cannot be determined from tumor positivity alone. Accordingly, any assertion of a wide therapeutic window is premature and must remain conditional on prospective quantification of tumor-to- normal uptake ratios via integrin αvβ6-targeted PET imaging, organ-level dosimetry, and toxicity monitoring. In the current study, adjacent non-neoplastic gastric mucosa served solely as an internal positive control and was not systemically scored; this lack of quantitative mucosal evaluation represents a key limitation that directly affects the interpretation of therapeutic feasibility. In our study, integrin αvβ6 expression was found in 92.5% of patients, including those with aggressive tumors that typically do not have additional targeted therapeutic options. Therefore, the results suggest that integrin αvβ6 may serve as a useful, broadly expressed biomarker.

A central translational question is how tumor microarchitecture influences integrin αvβ6-targeted radionuclide therapy. The present findings directly support three observations: predominantly membranous integrin αvβ6 localization, frequent expression across histological subtypes, and retained expression within signet-ring cells. This study was based exclusively on diagnostic biopsies obtained during gastroscopy. Therefore, the observed integrin αvβ6 staining pattern may not fully represent whole- tumor heterogeneity, and this limitation is particularly relevant when considering CLDN18.2 heterogeneity and theoretical integrin αvβ6- targeted radionuclide coverage.

Conceptually, intestinal-type tumors are usually highly cellular, with cohesive glandular, tubular, or solid tumor architecture and tumor-cell densities of approximately 50–90% of the involved tissue area [34], whereas poorly cohesive and signet-ring-cell carcinomas are more dispersed with a larger stromal fraction. β-emitting radionuclides such as Lutetium-177 (^177^Lu) and Terbium-161 (^161^Tb) can, in principle, produce a cross-fire effect that irradiates target-negative neighboring cells when integrin αvβ6- positive cells are spatially distributed throughout the tumor [35]. α-emitting radionuclides such as Actinium-225 (^225^Ac) have a much shorter effective tissue range and a higher linear energy transfer, than the β-emitting radionuclides, so their theoretical efficacy depends more on dense, homogenous target expression, potentially favoring compact, highly cellular tumors over dispersed ones [38,39]. In this study, there were no actual measurements of absorbed dose, radioligand uptake data, or biological response thresholds. However, the estimates listed in this paper are to be considered the theoretical minimum coverages under ideal assumptions, and, therefore, should not serve as validated therapeutic thresholds.

An important consideration for the clinical translation of integrin αvβ6-targeted radiotheranostics is the potential for on-target, off-tumor radiation exposure. Although integrin αvβ6 expression is largely absent from most quiescent adult tissues and remains relatively restricted under physiological conditions, expression has been documented in selected epithelial compartments, including the gastric mucosa, particularly during epithelial turnover, tissue remodeling, and wound healing. Furthermore, physiologic uptake of αvβ6- targeted PET tracers has been observed in structures such as the pituitary gland and choroid plexus, although the biological basis and clinical significance of this uptake remain incompletely understood. These findings suggest the existence of a potentially favorable therapeutic window while simultaneously emphasizing the need for comprehensive evaluation of normal-organ radiation exposure.

This study has several notable strengths that reinforce the validity of our findings and their translational significance. First, to the best of our knowledge, this is the first systematic immunohistochemical assessment of integrin αvβ6 expression that focuses specifically on untreated primary gastric and esophageal adenocarcinomas classified according to the fifth edition of the WHO classification of gastrointestinal tumors. This histological subtype allowed us to evaluate expression patterns within clinically relevant morphological subgroups, particularly poorly cohesive cell carcinomas and ring-shaped carcinomas. Second, the inclusion of paired comparative analyses with HER2 and CLDN18.2 within the same tumor samples represents a major methodological strength. Unlike studies comparing prevalence rates across different histological cohorts or heterogeneous patient groups, our direct case-level comparisons provide strong evidence for the broader expression of integrin αvβ6 for specific therapeutic targets within the same biological samples. This paired design minimizes inter-patient variability and strengthens the conclusion that αvβ6 fills a genuine diagnostic gap. Third, the usage of a standardized immunohistochemistry protocol with a well-characterized antibody and automated staining machine reduced the interassay variability and ensured reproducibility of the staining across different pathology facilities worldwide. Therefore, this practical, cost-effective solution does not depend on using specialized molecular platforms (such as next-generation sequencing or liquid biopsies), thus allowing for the development of immunohistochemistry as an accessible and high-throughput screening tool for selecting patients to participate in clinical trials and routine patient care.

This study has several limitations. It is retrospective and includes a relatively small cohort from archived pathology material. The cohort was derived only from a single institution and is enriched for high-grade, diffuse-type, and signet-ring-cell carcinomas. Another limitation is that integrin αvβ6 IHC scoring was done by a single experienced pathologist, without assessment of inter-observer or intra-observer reproducibility. Single-reader scoring has been applied in exploratory studies of immunohistochemical biomarkers, but reproducibility testing should precede successful implementation of integrin αvβ6 IHC as a histopathological screening tool. This is particularly relevant as mixed stain intensity was recorded with an intermediate intensity value to maintain some scoring precision. Subsequent validations in future studies must incorporate multi-reader, independent assessment and report agreement with clinically relevant measures, such as weighted κ for binary expression groups and intraclass correlation coefficients for continuous H-score values. Furthermore, the intensity and H-score of integrin αvβ6 in matched normal gastric mucosa should be evaluated in future studies. Survival data, treatment outcomes, and metastatic tissue were not available for comprehensive outcome correlation. This study used exclusively diagnostic gastric biopsy specimens obtained during gastroscopy; therefore, biopsy samples may not fully represent whole-tumor heterogeneity, particularly for CLDN18.2 and integrin αvβ6. The analysis focused on histopathology and immunohistochemistry and did not include αvβ6 PET imaging, autoradiography, ligand-binding assays, or absorbed-dose calculations. Finally, the discussion of radionuclide selection is conceptual and based on tumor architecture and particle range; it should not be interpreted as a validated therapeutic threshold.

## 5. Conclusions

In summary, our findings demonstrate that integrin αvβ6 is broadly expressed in gastric and gastroesophageal junction adenocarcinoma. While any detectable IHC staining was observed in 92.5% of cases, the therapeutically more relevant moderate to strong expression was present in 50.9% of cases and in 56.3% (9 of 16) of signet-ring-cell-containing tumors that frequently lack established therapeutic targets. Within the signet-ring-cell component itself, moderate-to-strong staining was present in 50.0% (8 of 16). In this cohort of 53 cases, integrin αvβ6 showed broader expression than HER2 and clinically relevant CLDN18.2, supporting its potential value as a complementary histopathology-assessable biomarker. The predominantly membranous localization of integrin αvβ6 provides a strong biological rationale for αvβ6-targeted molecular imaging and radioligand therapy. However, we emphasize that the H-score threshold that ensures adequate therapeutic efficacy has not been established.

β-emitting radionuclides such as ^177^Lu and ^161^Tb may be especially useful in tumors with heterogeneous expression because of the cross-fire effect, whereas α-emitting radionuclides such as ^225^Ac may be best suited to tumors with dense, spatially widespread target expression. Potential on-target, off-tumor exposure of gastric mucosa and other normal tissues must be evaluated carefully.

Overall, these findings establish integrin αvβ6 as a promising diagnostic and therapeutic target candidate in gastric cancer and justify further translational studies combining immunohistochemistry, αvβ6-targeted PET imaging, digital pathology, dosimetry, and therapeutic response assessment.

## Figures and Tables

**Table 1:** Clinicopathological characteristics and biomarker expression in 53 gastric/gastroesophageal adenocarcinoma cases.

Case No.	Site	Diagnosis/Grade	Signet-Ring-Cell Component	HER2 IHC Score	CLDN18.2 Expression	Integrin αvβ6 Expression in Tumor Cells	Integrin αvβ6 Expression in Signet-Ring Cells
1	Corpus	Adenocarcinoma G3	Present	0	+<5%	+10% (H-score 10, weak)	+5%
2	AEG II	Adenocarcinoma G2	Absent	0	+++80%	++50% (H-score 100, weak)	-
3	Corpus/antrum	Adenocarcinoma G3	Present	0	Not assessable	0% (H-score 0, negative)	0%
4	AEG II	Adenocarcinoma G2	Absent	0	Not assessable	++50% (H-score 100, weak)	-
5	Corpus	Adenocarcinoma G2	Absent	0	++75%	++30% (H-score 60, weak)	-
6	Cardia	Adenocarcinoma G3	Absent	0	+5%	++/+++80% (H-score 200, moderate)	-
7	Cardia	Adenocarcinoma G3	Absent	1+	0%	+++100% (H-score 300, strong)	-
8	Cardia	Adenocarcinoma G2	Absent	0	0%	++/+++100% (H-score 250, strong)	-
9	Cardia	Adenocarcinoma G3	Absent	Not assessable	Not assessable	++/+++100% (H-score 250, strong)	-
10	Corpus	Adenocarcinoma G3	Present	0	Not assessable	++/+++80% (H-score 200, moderate)	++75%
11	AEG II	Adenocarcinoma G2	Absent	3+	Not assessable	+50% (H-score 50, weak)	-
12	Pylorus	Adenocarcinoma G2	Absent	3+	Not assessable	++/+++90% (H-score 225, strong)	-
13	Antrum	Adenocarcinoma G3	Absent	0	0%	+10% (H-score 10, weak)	-
14	Corpus	Adenocarcinoma G2	Absent	0	Not assessable	+10% (H-score 10, weak)	-
15	Corpus	Adenocarcinoma G3	Present	0	0%	++80% (H-score 160, moderate)	++80%
16	Antrum	Adenocarcinoma G2	Absent	0	Not assessable	+10% (H-score 10, weak)	-
17	Corpus/antrum	Adenocarcinoma G2	Absent	1+	0%	++/+++100% (H-score 250, strong)	-
18	AEG II	Adenocarcinoma G2	Absent	0	0%	++/+++50% (H-score 125, moderate)	-
19	Antrum	Adenocarcinoma G3	Present	0	Not assessable	++50% (H-score 100, weak)	++50%
20	Antrum	Adenocarcinoma G2	Absent	0	Not assessable	+5% (H-score 5, weak)	-
21	Antrum	Adenocarcinoma G3	Absent	0	Not assessable	+10% (H-score 10, weak)	-
22	Corpus	Adenocarcinoma G2	Absent	0	Not assessable	++90% (H-score 180, moderate)	-
23	Corpus	Adenocarcinoma G3	Present	0	Not assessable	+5% (H-score 5, weak)	+5%
24	AEG II	Adenocarcinoma G3	Absent	0	Not assessable	++/+++30% (H-score 75, weak)	-
25	AEG II	Adenocarcinoma G3	Absent	0	Not assessable	+++50% (H-score 150, moderate)	-
26	AEG I	Adenocarcinoma G3	Absent	0	0%	0% (H-score 0, negative)	-
27	Corpus	Adenocarcinoma G3	Present	0	0%	++75% (H-score 150, moderate)	++75%
28	Corpus	Adenocarcinoma G3	Present	0	0%	+++100% (H-score 300, strong)	+++100%
29	Corpus	Adenocarcinoma G3	Absent	3+	0%	++/+++40% (H-score 100, weak)	-
30	Corpus	Adenocarcinoma G2	Absent	0	0%	++75% (H-score 150, moderate)	-
31	Corpus	Adenocarcinoma G3	Present	0	0%	++30% (H-score 60, weak)	++30%
32	Corpus/antrum	Adenocarcinoma G3	Absent	0	+++80%	+++100% (H-score 300, strong)	-
33	Corpus	Adenocarcinoma G3	Present	0	0%	++/+++95% (H-score 237.5, strong)	++/+++95%
34	Corpus	Adenocarcinoma G3	Absent	0	+40%	+2% (H-score 2, weak)	-
35	Antrum	Adenocarcinoma G3	Present	0	+30%	++75% (H-score 150, moderate)	++75%
36	Antrum	Adenocarcinoma G3	Absent	0	0%	++/+++95% (H-score 237.5, strong)	-
37	AEG II	Adenocarcinoma G3	Absent	1+	0%	++20% (H-score 40, weak)	-
38	Antrum	Adenocarcinoma G3	Present	1+	++/+++80%	+/++75% (H-score 112.5, moderate)	+/++75%
39	Corpus	Adenocarcinoma G3	Absent	1+	++/+++40%	+5% (H-score 5, weak)	-
40	Antrum	Adenocarcinoma G3	Absent	0	+20%	++75% (H-score 150, moderate)	-
41	Antrum	Adenocarcinoma G2	Absent	0	0%	++100% (H-score 200, moderate)	-
42	Corpus	Adenocarcinoma G3	Present	0	++80%	+/++50% (H-score 75, weak)	+/++50%
43	Antrum	Adenocarcinoma G3	Absent	0	0%	++100% (H-score 200, moderate)	-
44	Antrum	Adenocarcinoma G3	Absent	0	++100%	+/++50% (H-score 75, weak)	-
45	AEG II	Adenocarcinoma G2	Absent	0	0%	+30% (H-score 30, weak)	-
46	Antrum	Adenocarcinoma G3	Present	0	0%	+5% (H-score 5, weak)	0%
47	AEG II	Adenocarcinoma G3	Absent	0	0%	0% (H-score 0, negative)	-
48	Corpus	Adenocarcinoma G3	Present	0	0%	++/+++90% (H-score 225, strong)	+90%
49	AEG II	Adenocarcinoma G2	Absent	2+, FISH negative	3%	++/+++100% (H-score 250, strong)	-
50	Corpus	Adenocarcinoma G3	Present	0	10%	++/+++90% (H-score 225, strong)	++/+++90%
51	Corpus	Adenocarcinoma G2/G3	Absent	0	++90%	0% (H-score 0, negative)	-
52	Esophagojejunal anastomosis	Adenocarcinoma G3	Absent	0	+10%	++/+++90% (H-score 225, strong)	-
53	Corpus/antrum	Adenocarcinoma G2	Absent	3+	+30%	++/+++75% (H-score 187.5, moderate)	-

AEG—adenocarcinoma of the esophagogastric junction; CLDN18.2—claudin 18.2; FISH—fluorescence in situ hybridization; HER2—human epidermal growth factor receptor 2; H-score—histochemical score. Staining intensity: +—weak; ++—moderate; +++—strong. H-score categories: negative = 0; weak = 1–100; moderate = 101–200; strong = 201–300. Percentages indicate the approximate proportion of positive tumor cells in the evaluated compartment. For the CLDN18.2 column, entries given as a percentage alone (without an accompanying intensity symbol) indicate that staining intensity was not specified in the original diagnostic report, whereas entries with symbols (+/++/+++) include the recorded intensity. “Not assessable” denotes that CLDN18.2 IHC could not be performed due to tissue exhaustion on the diagnostic block, which precluded additional sectioning. For HER2, IHC scores are reported per standard gastric cancer guidelines (0, 1+, 2+, 3+); equivocal (2+) cases undergo reflex FISH testing to assess gene amplification, and the final combined designation is shown in the table (e.g., case 49: 2+ IHC with negative FISH, classified as HER2-negative overall).

**Table 2:** Summary of therapeutic biomarker expression profiles (HER2, CLDN18.2 and integrin αvβ6) and positivity rates in the gastric and gastroesophageal junction adenocarcinoma cohort (*n* = 53).

Biomarker	Positive Cases	Percentage	Key Observation
HER2 positive	4/53	7.5%	Lower than published international rates
CLDN18.2 any expression	17/38 assessable	44.7%	Moderate overall expression
CLDN18.2 high expression ≥ 75%	7/38 assessable	18.4%	Lower than SPOTLIGHT/GLOW screening rates
Integrin αvβ6 positive	49/53	92.5%	Most frequently expressed marker
Integrin αvβ6 moderate/strong	27/53	50.9%	Broad expression across cohort
Integrin αvβ6 in signet-ring cells	14/16	87.5%	Frequently retained in signet-ring-cell component

## Data Availability

The data presented in this study are available from the corresponding author upon reasonable request.
